# Supplementary material for: SNX5 promotes antigen presentation in B cells by dual regulation of actin and lysosomal dynamics
Source: Life Sci Alliance. 2024 Oct 24;8(1):e202402917. doi: 10.26508/lsa.202402917 (PMC11502673; doi:10.26508/lsa.202402917)
Supplement: Supplementary file 3 [file LSA-2024-02917_SdataF2_FS1.pdf]

Fig 2D: Synaptic membrane

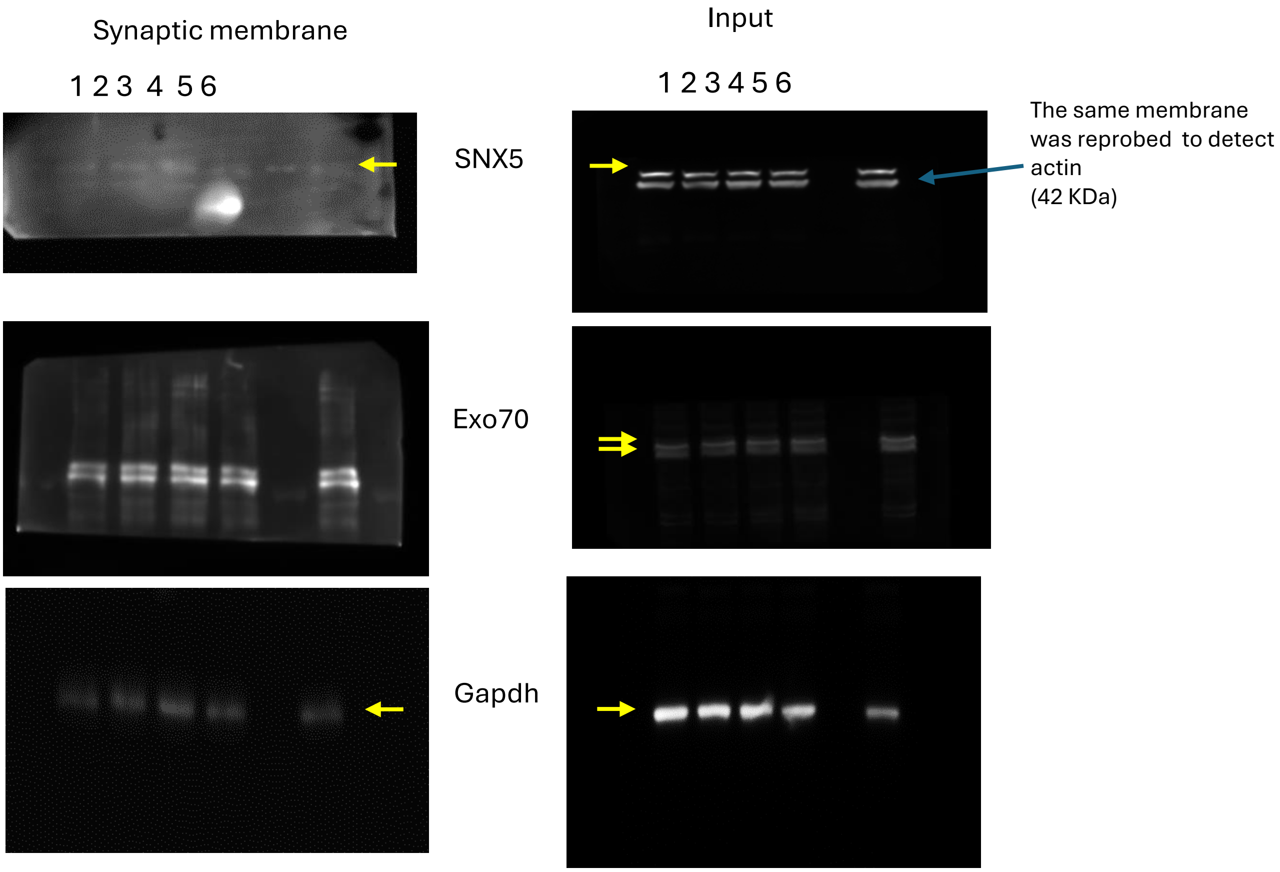

1: 0 min
2: 30 min
3: 60 min
4: 120 min
5: Std
6: Total lysated

Fig 2D: Synaptic membrane n2

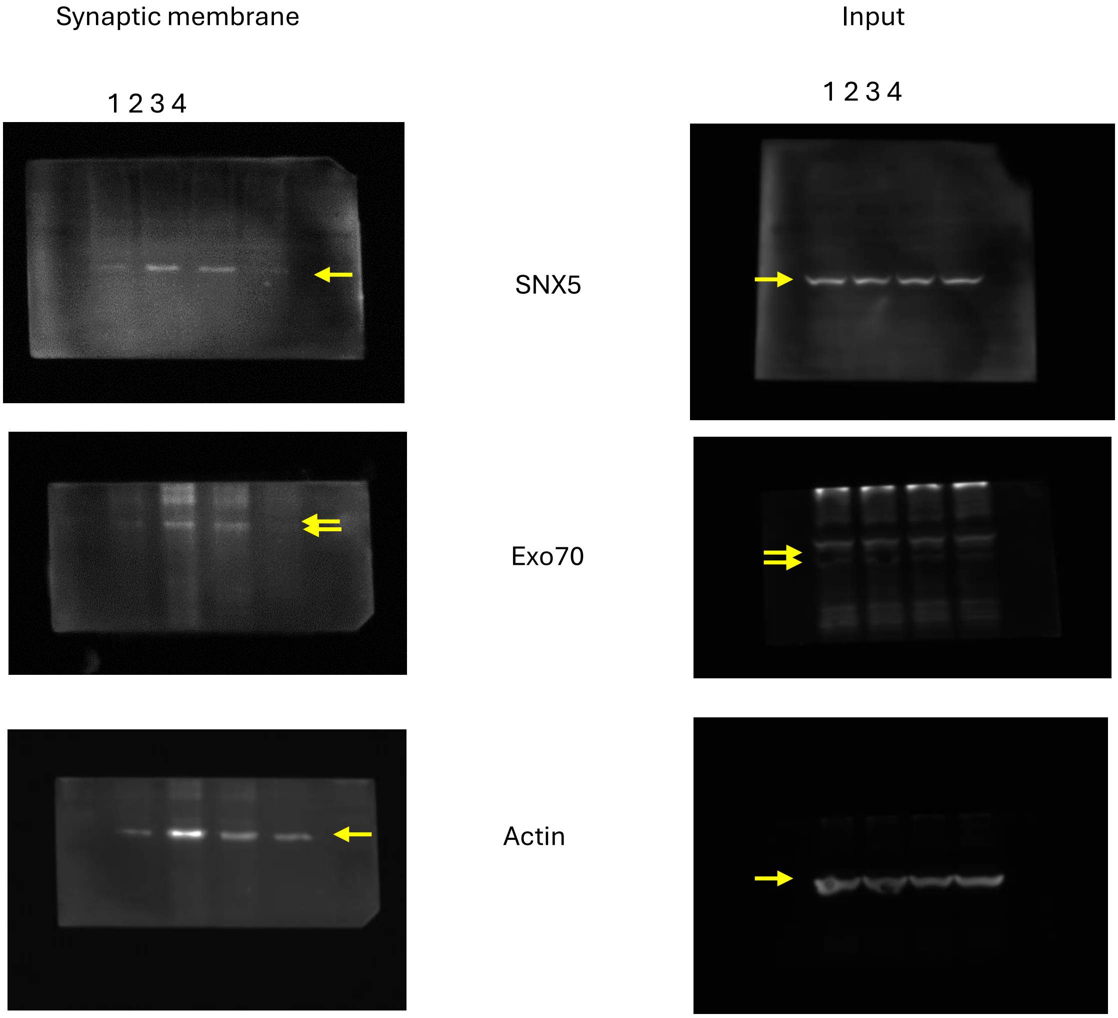

1: 0 min
2: 30 min
3: 60 min
4: 120 min

Figure 1SA
SNX5 silencing in B cells

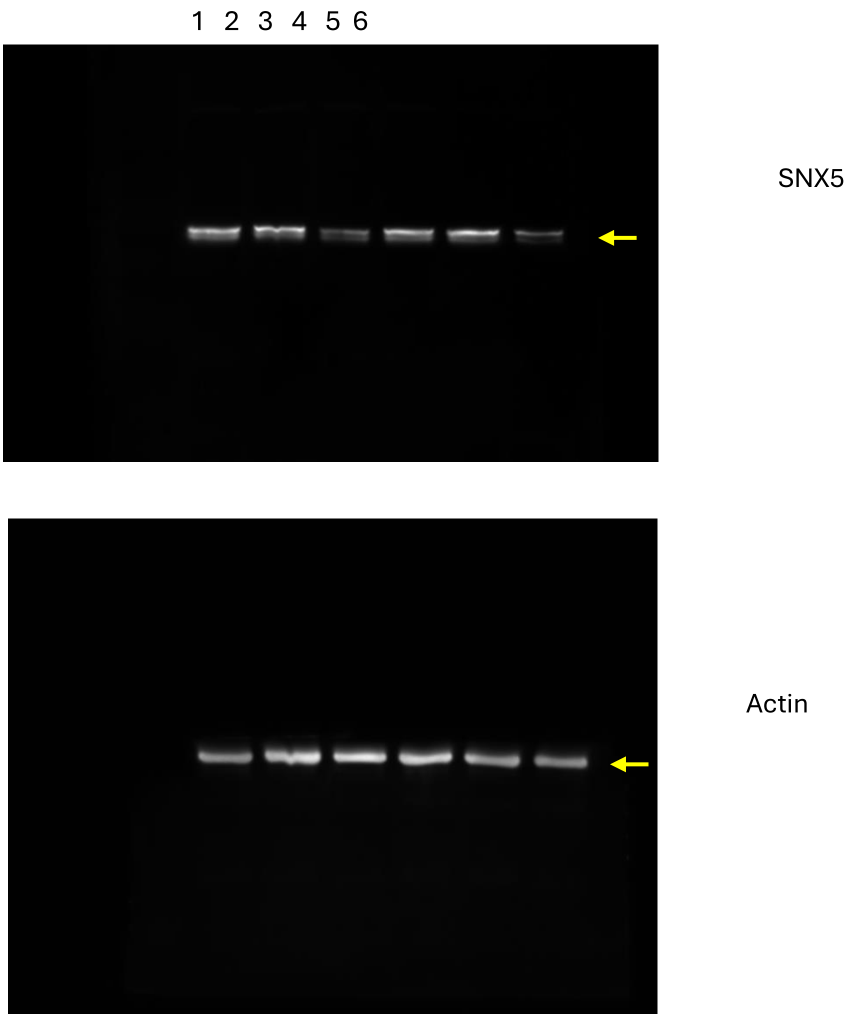

1: siCtrl
2: siSNX5-A
3: **siSNX5-B**
4: siCtrl
5:siSNX5-A
6: **siSNX5-B**

Figure 1SA
SNX5 silencing in B cells

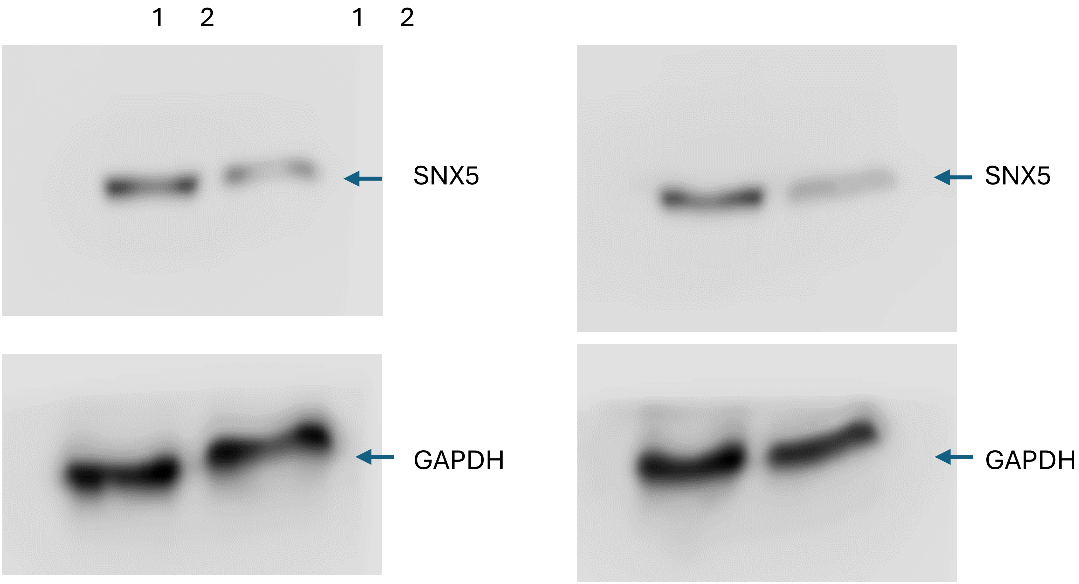

1: siCtrl

2: siSNX5-B
